# Editorial for the Special Issue on “Multidisciplinary Insights on Bone Healing”

**DOI:** 10.3390/biology11121776

**Published:** 2022-12-07

**Authors:** Alexandre Anesi, Francesco Cavani

**Affiliations:** 1 Department of Medical and Surgical Sciences for Children and Adults, Cranio-Maxillo-Facial Surgery, University of Modena and Reggio Emilia, Largo del Pozzo 71, 41125 Modena, Italy; 2 Department of Biomedical, Metabolic and Neural Sciences, Section of Human Morphology, University of Modena and Reggio Emilia, Largo del Pozzo 71, 41125 Modena, Italy

Animal and human bone damage can be considered differently according to a macro- or micro-level analysis. In a clinical setting, bone loss and damage are often macroscopically described as bone defects. Primary and secondary bone loss can be characterized in clinical practice. Primary bone loss may arise in bone diseases, such as osteopenia and malignancy. Secondary bone loss is most commonly caused by metastatic disease, while trauma is the most common cause of macroscopic bone defects. Moreover, in dentistry and in maxillo-facial surgery, the bone loss of the alveolar bone after dental extraction is defined as bone atrophy [1,2]. On the other hand, micro-level investigations on bone damage can shed further light on radiologic and clinical descriptions. Scanning electron microscopy (SEM) evaluation and micro-mechanics in bone tissue have revealed the presence of microcracks related to the compression of bone, excessive bone strain [3,4,5], and also to physiological loading conditions and under excessive loads [6]. Bone overheating during the bone cutting process is a further well-known risk factor for microscopic bone injury [7].

Both of the above-described macro- and micro-damages to bone tissue activate the bone healing process. Bone healing is a sophisticated multifactorial “system” composed of both macro- and microscopic agents that interact with each other in order to obtain a recovery of the correct morphological and functional features [8]. Nonetheless, this complex process does not always “work” (i.e., to restore the original function and morphology of wounded or lost bone); therefore, a lot of research is still underway in order to find ways to improve bone healing in circumstances where it is difficult or hindered. A continuous cooperation between basic laboratory investigations and clinical observations, from the fields of biology to regenerative medicine, from engineering to physics, help fulfill this goal. This Special Issue, entitled “Multidisciplinary Insights on Bone Healing”, contains five papers which span from the biophysical stimulation of bone repair to the use of natural or synthetic materials to stimulate bone deposition. This Special Issue comprises:

- An experimental study [9] aiming to characterize 3D polycaprolactone (PCL) scaffolds reinforced with a novel Mg-doped bioactive glass (Mg-BG) characterized by good mechanical properties and biological reactivity. Two different polymer-to-particles weight ratios were tested for physical characteristics and biological in vitro activity/toxicity. Compared to pure PCL, the 50/50 wt% formulation showed high mechanical resistance and good biocompatibility, bioactivity, and cell adhesion; therefore, the use of the composite PCL/Mg-BG scaffolds might be able to promote cell viability and support mechanical loading in the host trabecular bone.
- A review focuses on the stimulation of Pulsed Electromagnetic Fields (PEMFs) [10] in animal models of bone alterations. It has been shown that PEMFs are able, using certain signal characteristics and treatment times, to improve bone regeneration and prevent bone loss. In vivo investigations on PEMF stimulation are reviewed, focusing on molecular and morphological improvements in bone in order to better understand the biological mechanism of PEMF and its effect on bone healing so that each researcher/clinician might choose the most appropriate signal for a specific bone disorder in order to obtain a specific result.
- A wide narrative review [2] aims to identify the best approach to treat peri-implant diseases that usually lead to bone loss around dental implants, causing implant failure. Despite many investigations aimed at identifying the best approach to treat these conditions, there is still no universally recognized protocol to solve these complications successfully and predictably. Still, the clinician dealing with such pathologies has to face the following questions: Is any product superior to the other? Should a membrane be added to the graft? Is any method of decontamination superior? Therefore, the authors review recent studies on peri-implant regeneration and their outcomes, as well as background studies that led to the current knowledge of materials and techniques, in order to try to shed some light on the topic.
- A systematic review [11] on 20 selected papers illustrates the use of avian eggshell as a bone regeneration material, since it has been shown that it is a biocompatible grafting material with bone formation capabilities. It can be combined with other materials to enhance its osteoconductive and regenerative properties. Eggshell is a promising biomaterial to be used in bone grafting procedures, although further research is needed.
- A systematic review [12] highlights the role of photobiomodulation (PBM) on in vivo bone healing, in particular in the management of socket preservation. PBM is a technique that employs photons at the red and infrared wavelengths that can interact with specific photoreceptors located within the cell, modifying cellular metabolism by increasing mitochondrial ATP production. PBM has been shown in previous studies to modulate tissue inflammation, stimulate growth factor expression and cell proliferation, and accelerate the healing processes. In conclusion, the review shows that, when irradiated using the appropriate parameters, PBM could improve osteoproliferation and osteoinduction for socket preservation in healthy and sick animal models and human subjects, as well as in the presence or not of an allograft or biomaterial.
